# Associations between the Antioxidant Network and Emotional Intelligence: A Preliminary Study

**DOI:** 10.1371/journal.pone.0101247

**Published:** 2014-07-01

**Authors:** Mirko Pesce, Maria R. Sergi, Alessia Rizzuto, Raffaella Tatangelo, Marco Tommasi, Laura Picconi, Michela Balsamo, Valentina Gatta, Liborio Stuppia, Alexander B. Siegling, Elif Gökçen, Alfredo Grilli, Aristide Saggino

**Affiliations:** 1 Department of Psychological, Humanistic and Territorial Sciences University G. d'Annunzio, Chieti, Italy; 2 London Psychometric Laboratory, University College London, London, United Kingdom; University of South Florida College of Medicine, United States of America

## Abstract

**Background:**

Emotional intelligence (EI) can be broadly defined as the ability to cope with environmental demands. In the scientific research, however, there is not a univocal precise definition of EI and recent articles have underlined the necessity to explore its biological basis to advance understanding of the construct. The aim of study was to investigate if the antioxidant network may be associated with typical-performance or trait EI.

**Methods:**

The study group consisted of 50 women (age, M = 25.10, SD = 3.87). Super Oxide Dismutase (SOD), Catalase (CAT), Glutathione Reductase (GR), and Glutathione Peroxidase (GPx) activities were evaluated on proteins extracted from Peripheral Blood Mononuclear Cells. Participants completed the Italian version of the EQ-i (Bar-On, 1997) as a measure of trait EI.

**Results:**

We observed positive and significant correlations between some biological variables and EQ-i scores, and a significant predictive effect of CAT activity when controlling for related biological variables, age, and smoking.

**Conclusions:**

Our preliminary study suggests that the antioxidant network may constitute some of trait EI's biological basis. In particular, CAT and the SOD/CAT ratio could be two biological variables involved in some specific components of EI.

## Introduction

Scientific landscape shows a great attention for emotional intelligence (EI). However, the definition of this construct remains somewhat elusive, with a number of competing models emerging in the literature in recent decades [1]–[7]. Bar-On's model is one of the most prevalent models in the literature, comprising a global trait EI dimension, five broad factors, and 15 more narrow facets [8]. It broadly conceptualizes EI as “an array of non-cognitive capabilities, competencies, and skills that influence one's ability to succeed in coping with environmental demands and pressures” (p. 14). However, the measure based on this model, the Emotional Quotient Inventory (EQ-i), is most appropriately viewed as a measure of trait EI, since it assesses typical rather than maximum performance [3]. Trait EI is an umbrella construct that integrates the range of personality traits linked to emotions; it represents the affective dimension of personality [3].

Regardless of whether the focus is on the trait or ability dimension, a challenge with EI is to determine the set of measurable manifestations, which explains the large quantity of models that have emerged over the years. A critique levelled against Bar-On's model is that it is over-inclusive, comprising a large quantity of social and emotional skills that determine how we understand ourselves and others, how we relate to others, and how we cope with daily activities [8]. In particular, it has been suggested that some of these factors are too broad and conceptually unrelated to EI [9]–[11]. In order to improve our understanding of this construct, researchers have emphasized the necessity to explore its biological basis [12]–[15]. To date, however, the role of biological factors has not been examined across the EI literature [9], [14], [16]. The present study provides a starting point for research in this promising area, seeking to identify biological factors implicated in typical-performance or trait EI.

Biological factors potentially related to EI are the molecules involved in the regulation of oxidative and antioxidant processes, which were found to play an important role in neuropsychological diseases characterized by cognitive impairment [17]–[19], in elicitation of mood types, such as anxiety in healthy women [20], and in depression among clinical patients [21]. Under normal physiological conditions, a balance is maintained between oxidative and antioxidant systems. Super Oxide Dismutase (SOD), Catalase (CAT), Glutathione Reductase (GR) and Glutathione Peroxidase (GPx) exert a key function in the antioxidant network and the activities of these enzymes protect cell from the harmful excess of Reactive Oxygen Species [22].

In addition, the reduction of the enzymatic antioxidant activity has been associated with impairment of cognitive performances at both the central nervous system and the peripheral blood levels [19], [23]. The reduction of serum level of SOD activity is associated with a distressed personality, characterized by the tendency to experience prevalently negative emotions and to inhibit the expression of these emotions in a social context [24]. Further, serum SOD and GPx activities of anxious patients were recently found to be significantly lower than those of controls [25].

The purpose of this study was to investigate if the antioxidant network (i.e., SOD, CAT, GR and GPx activities and their ratio in Peripheral Blood Mononuclear Cells) may be implicated in trait EI. The relationships of these biological variables with the trait EI scores of healthy subjects were examined. Research on emotions [26] and EI specifically has revealed gender differences across several emotion-related attributes, although at the global construct level these differences tend to average out in the case of trait EI, which is the focus of this study (for a more comprehensive discussion of gender differences in EI, see reference [27]. To control any potential confounding effects linked to gender differences, this preliminary study was conducted on a female-only sample.

## Method

### Participants

The participants were an opportunity sample of fifty female psychology students. Their ages ranged from 19 to 39 years (*M* = 25.10, *SD* = 3.88). Written informed consent was obtained from all participants before starting the experiment, according to the Declaration of Helsinki. The study was approved by the Ethical Committee of the “G. d'Annunzio” University of Chieti-Pescara, Italy.

Participants were in good physical health, not taking medication, and had no history of psychiatric or somatic disorders. The average BMI based on 49 participants was within a normal range (*M* = 22.00, *SD* = 3.73) and eighteen participants identified themselves as smokers and the rest as non-smokers. The C-reactive protein serum level was measured as a nonspecific marker for inflammation and utilized as an exclusion criterion [28], [29]. However, all students recruited did not exceed a C-reactive protein level of 5 mg/L at the time of blood collection and therefore none were excluded.

### Measures and procedure

#### EI

The EI of each subject was assessed using the Italian version of the EQ-i [8] a 133-item self-report inventory based on Bar-On's model. The items are responded to on a 5-point Likert scale ranging from 1 (*very seldom true or not true for me*) to 5 (*very often true of me or true of me*). The EQ-i items produce a total EI score, five composite scores, and 15 facets scores. The composites are Intrapersonal EI, Interpersonal EI, Adaptability, Stress Management, and General Mood; the 15 facets are Emotional Self-Awareness, Assertiveness, Self-Regard, Self-Actualization, Independence, Empathy, Interpersonal Relationship, Social Responsibility, Problem-Solving, Reality-Testing, Flexibility, Stress Tolerance, Impulse Control, Optimism and Happiness.

### Biological variables

#### Sample collection

At the same time of EI assessment, venous blood was collected by phlebotomy in EDTA vacutainers (6 mL K_2_EDTA, Becton Dickinson, Franklin Lakes, NJ, USA) and processed within 2 hours of procurement. Peripheral Blood Mononuclear Cells were isolated by density-gradient centrifugation through Ficoll/Hypaque (Pharmacia, Piscataway, NJ, USA) from the blood of the students. Protein extractions were performed as previously described [30].

#### Cu, Zn-SOD activity

SOD activity was determined as described by Sun and Zigman [31]. The assay mixture contained 50 mM sodium carbonate buffer, pH 10, epinephrine 0.1 mM (Sigma), and protein extract (10 mg) in a final volume of 2.5 ml. The inhibitory effect of SOD on the autoxidation of epinephrine, with the use of 1.25 mM KCN to discriminate the CN^−^ insensitive MnSOD from the CN^−^ sensitive Cu, ZnSOD was assayed spectrophotometrically at 480 nm at 25°C. Percentage inhibition values were converted into activities by using a purified Cu, Zu bovine SOD as standard (Sigma).

#### CAT activity

CAT activity was measured spectrophotometrically [32]. The decomposition of H_2_O_2_ was monitored continuously at 240 nm. The assay mixture in a final volume of 3 ml contained 10 mM potassium phosphate buffer, 10 mM H_2_O_2_ and 5 mg of protein of enzymatic extract. CAT units were defined as 1 mole H_2_O_2_ decomposed/min at 25°C.

#### GPx and GR activity

Quantification of GPx activity was evaluated as previously described [33]. Briefly, the activity of the Se-dependent GSH peroxidise was measured with H_2_O_2_ (0.25 mM) as substrate. The oxidation of NADPH was followed at 25°C on a Hewlett and Packard spectrophotometer at 340 nm. One unit was defined as 1 mmol of GSH oxidized min.

GR activity was specrophotometrically monitored at 340 nm and 25°C [34]. The assay mixture in a final volume of 3 ml contained 0.1 mM potassium phosphate buffer, ph 7.4, 1 mM EDTA, 1 mM GSSG (Sigma), 0.16 mM NADPH (Sigma) and 1–30 microg of protein of Peripheral Blood Mononuclear Cells. One unit of enzyme activity was defined as 1 mmol of NADPH oxidized/min at 25°C.

### Statistical analysis

Pearson correlation coefficients (*r*) were computed to examine the relationships among the study variables. We previously analyzed the linearity of correlation through the examination of scatterplots among CAT and EQ-i scores (Supplementary file S1). Further, the EQ-i total score was regressed on biological variables to examine the coexisting effects of these biological variables on EI. All statistical tests were evaluated at an alpha level of .05. Statistical analysis was performed using SPSS ®Advanced Statistical 18.0.1 software (SPSS Inc, Chicago, Illinois, USA).

## Results

Descriptive statistics for the biological variables and EQ-i scale scores are shown in Tables 1 and 2, respectively. Table 2 also shows the internal reliabilities for the EQ-i scores, which were mostly within an acceptable range.

**Table 1:** Descriptive Statistics for Biological Variables.

Variable	*M*	*SD*
SOD (U/mL)	27.08	14.53
CAT (U/mL)	1.52	1.18
SOD/CAT	58.44	141.47
GR (U/mL)	21.23	9.18
GPx (U/mL)	9.75	4.88
GR/GPx	2.51	1.21

**Note**. *N* = 48–50. SOD  =  Super Oxide Dismutase; CAT  =  Catalase; GR  =  Glutathione Reductase; GPx  =  Glutathione Peroxidase.

**Table 2:** Descriptive Statistics and Internal Reliabilities for EQ-i Scores.

Scale	*M*	*SD*	Cronbach's α
RAeq	155.66	23.13	.95
ES	30.24	5.98	.88
AS	26.52	4.89	.79
SR	35.28	6.52	.91
SA	37.66	5.75	.84
IN	25.96	4.94	.80
EReq	116.86	11.24	.82
EM	31.72	3.15	.45
IR	44.38	6.16	.81
RE	40.76	4.44	.65
ADeq	96.80	12.49	.86
PS	31.78	4.74	.76
RT	35.90	6.27	.78
FL	29.12	4.78	.78
SMeq	63.86	10.36	.87
ST	31.42	5.80	.83
IC	32.44	6.12	.82
GMeq	68.10	9.70	.91
HA	36.84	5.61	.84
OP	31.26	4.76	.85
EQ-i tot	501.28	57.70	.97

**Note.** *N* = 50. EQ-i tot  =  Emotional Quotient Inventory total score; RAeq  =  Intrapersonal; EReq  =  Interpersonal; ADeq  =  Adaptability; SMeq  =  Stress Management; GMeq  =  General Mood; ES  =  Emotional Self-Awareness; AS  =  Assertiveness; SR  =  Self-Regard; SA  =  Self-Actualization; IN  =  Independence; EM  =  Empathy; IR  =  Interpersonal Relationship; RE  =  Social Responsibility; PS  =  Problem- Solving; RT  =  Reality-Testing; FL  =  Flexibility; ST  =  Stress Tolerance; IC  =  Impulse Control; HA  =  Happiness; OP  =  Optimism.

Preliminary analyses showed that age (*r* = .22), smoking (*r* = .22), and BMI (*r* = .24) were all unrelated to participants' EQ-i total scores (*p*>.05). When considering the five composites, however, age correlated significantly with Adaptability (*r* = .22, *p* = .01), whereas smoking (*r* = .31, *p* = .01) and BMI (*r* = .33, *p* = .01) both correlated significantly with Stress Management.

Table 3 shows the bivariate correlations between the biological variables and EQ-i scores. CAT emerged as the single most salient predictor of EQ-i scores, correlating significantly with the EQ-i total score and three of its five composites scales: Adaptability, Stress Management, and General Mood. Further, CAT correlated significantly with six of the 15 facets: Self-Regard, Reality-Testing, Stress Tolerance, Impulse Control, Happiness, and Optimism. None of the other biological variables correlated significantly with either the total EQ-i score or the composites. Only the SOD/CAT ratio was significantly associated with one of the 15 facets (Social Responsibility).

**Table 3:** Bivariate Correlations between Biological Variables and EQ-i Scores.

Scale	SOD	CAT	SOD/CAT	GR	GPx	GR/GPx
RAeq	.10	.26	−.03	−.02	−.02	.16
ES	.16	.21	.01	−.17	−.09	.15
AS	.25	.08	.02	−.09	.06	.05
SR	.00	.34*	−.06	.08	.06	.16
SA	−.10	.24	.01	.10	−.06	.19
IN	.17	.17	−.12	−.05	−.05	.08
EReq	.02	.24	−.23	−.03	−.05	.13
EM	.05	.14	−.25	−.19	−.12	.08
IR	.10	.24	−.26	.01	.00	.13
RE	−.12	.17	−.33*	.04	−.03	.09
ADeq	.01	.29*	−.08	−.11	−.09	.06
PS	.05	.08	−.05	−.20	−.22	.05
RT	.09	.32**	−.06	.11	.07	.05
FL	−.14	.25	−.08	−.21	−.10	.04
SMeq	−.14	.44**	−.14	.12	.07	.01
ST	−.06	.39**	−.16	−.08	−.03	.07
IC	−.19	.38**	−.09	.27	.14	−.05
GMeq	−.07	.34*	−.13	.03	.05	.11
HA	−.07	.31*	−.05	.09	.09	.10
OP	−.07	.33*	−.21	−.03	.00	.09
EQ-i tot	.01	.35*	−.12	−.01	−.02	.12

*Note*. *N* = 48–50. SOD  =  Super Oxide Dismutase; CAT =  Catalase; GR  =  Glutathione Reductase; GPx  =  Glutathione Peroxidase; EQ-i tot  =  Emotional Quotient Inventory total score; RAeq  =  Intrapersonal; EReq  =  Interpersonal; ADeq  =  Adaptability; SMeq  =  Stress Management; GMeq  =  General Mood; ES  =  Emotional Self-Awareness; AS  =  Assertiveness; SR  =  Self-Regard; SA  =  Self-Actualization; IN  =  Independence; EM  =  Empathy; IR  =  Interpersonal Relationship; RE  =  Social Responsibility; PS  =  Problem- Solving; RT  =  Reality-Testing; FL  =  Flexibility; ST  =  Stress Tolerance; IC  =  Impulse Control; HA  =  Happiness; OP  =  Optimism.

*p<.05.

**p<.01.

Table 4 presents the multiple regression analysis results. EQ-i total score, was regressed on SOD, CAT, and SOD/CAT ratio in a simultaneous regression analysis, using age and smoking as control variables.

**Table 4:** Regression Analysis Summary for Biological Variables Predicting EQ-i Total Score.

Variable	*B*	*SE B*	β	*t*	*p*
Age	3.18	1.96	.21	1.62	.112
Smoking	30.87	15.64	.26	1.97	.055
SOD	.49	.58	.12	.83	.408
CAT	18.85	7.01	.38	2.69	.010
SOD/CAT	.00	.06	−.01	−.05	.959

*Note*. *N* = 50. *F*(5, 44) = 2.86, *p* = .03, *R* ^2^ = .25, *R* ^2^_Adj_ = .16. SOD  =  Super Oxide Dismutase; CAT  =  Catalase; EQ-i  =  Emotional Quotient Inventory.

BMI was not included here, as it was missing the data from one participant and to ascertain a sufficient number of cases per predictor. When adding BMI to the equation, CAT activity remained a significant predictor and no others became significant (Supplementary file S2).

CAT activity was the only significant predictor of the EQ-i total score (β = .38, *p* = .010), and the overall model was significant, *F*(5, 44) = 2.86, *p* = .03, accounting for 25% of the EQ-i variance.

## Discussion

The aim of the present study was to examine the associations between the activities of the major cellular antioxidant enzymes (SOD, CAT, GR, and GPx), their ratios (SOD/CAT and GR/GPx) and EI, as assessed by the EQ-i. Our data suggest that CAT activity is positively associated with EI. In particular, CAT activity correlated with the EQ-i factors of Adaptability, Stress Management, General Mood as well as with the EQ-i total score. These EQ-i scales are aligned to Bar-On's EI model, in which Adaptability is understood as the capacity to cope with social situations through flexible behaviour; Stress Management is linked to competence to manage the stress and the unpleasant life events; and General Mood represents a positive attitude toward the life. Furthermore, CAT correlated with EQ-i subscales of Self-Regard, Reality-Testing, Stress Tolerance, Impulse Control, Optimism, and Happiness. The predictive effect of CAT on the EQ-i total score, which is of main interest, remained significant after controlling for age, smoking, and the other biological variables.

The enzymes of the antioxidant network protect the cell from harmful concentration of Reactive Oxygen Species. In particular, CAT and GPx act sequentially to SOD, scavenging its product hydrogen peroxide. Again, GR acts reducing the oxidized form of Glutathione (GSSG) to reduced Glutathione (GSH), that exerts antioxidant action and also is involved in GPx activity. Then, lower SOD/CAT as well as GR/GPx ratio suggests a higher efficiency in antioxidant response. These reactions also occur during cellular metabolism. When the level of SOD activity increases in the cell without a proportional increment in peroxidases activity, the cell faces a peroxide overload challenge. Peroxide can react with transitional metals and generate the radical hydroxyl, which is the most harmful Reactive Oxygen Species [22]. Hence, the significant positive correlations between CAT activity and several EQ-i scales and sub-scales, as well as the negative correlation between SOD/CAT ratio and RE scores suggest a higher efficiency in scavenging Reactive Oxygen Species, characterized by more elevated EQ-i scores in women.

Our findings are in accordance with previous studies postulating that cognitive decline is related to inadequate antioxidant capacity and that perturbation in oxidative metabolism correlated with personality traits that negatively modulated EI [24], [35]–[37]. Of note, the significant and positive correlation that we observed specifically between CAT activity and the EQ-i scales of Adaptability and Stress management is in accordance with data underlining a disturbed balance between oxidant and antioxidant defence systems during psychological stress [38]. This imbalance leads to oxidative damage and influences tissue function and dysfunctions in many organs, including the brain [39], [40]. Therefore, it is reasonable to observe that ineffective management of stress could result in the disturbance of redox homeostasis in the body as a consequence of decreased activities of protective antioxidant enzymes. The decrease of CAT activity was reported in research on animals, in which mice exposed to psychological stress showed significantly lower CAT activity in muscular tissue relative to matched controls [41]. Again, imbalance in SOD and CAT activities, reported as increase in SOD/CAT ratio was already observed in hippocampus of adult rats, at the same time of anxious behaviour manifestation [42].

The positive correlation between CAT activity and the General mood scale is also consistent with previous findings on related constructs. In general, several reports suggest that lower antioxidant defences against lipid peroxidation exist in patients with depressed mood and that there is a therapeutic benefit from antioxidant supplementation in these patients [43]. In particular, it was already reported that within antioxidant network enzymes, only CAT activity was significantly reduced in erythrocytes of patients with affective disorders in both pre- and post-treatment periods compared to the control group [44].

Although rules of thumb for the minimum number of cases were met, a limitation of the current study is the modest sample size. Some correlations and beta weights were in excess of .20 but did not reach significance. Likely, these would reach statistically significant levels in larger samples. Nonetheless, the results of this preliminary study underline a relationship among the variables evaluated: CAT probably seem to be implicated in trait EI. Another limitation is that the effects of other relevant variables were not controlled. It remains to be investigated to what extent CAT activity is a unique predictor of trait EI, beyond higher-order personality factors, which share considerable variance with the construct. In fact, EI as assessed through typical-performance methods (i.e., trait EI) ought to be integrated within extant models of personality. Finally, future research in this area will inevitably need to examine males, who tend to differ from women across a range of emotion-related attributes subsumed under various EI constructs and may show different developmental trajectories in these qualities.

## Conclusion

The findings provide preliminary evidence that EI has some of its basis in antioxidant enzymatic activities that physiologically regulate oxidative status during cellular functioning. In sum, the relationship between EI and oxidative status warrants further investigation, including evaluation of other antioxidant enzymes, as well as other non-enzyme antioxidants, in order to better understand the biological underpinnings of EI.

## Supporting Information

File S1 **Scatterplots among CAT activity and EQi scores.**
(DOC)

File S2 **Biological and psychometric raw data analysis.**
(XLS)
