# Supplementary material for: Associations between the Antioxidant Network and Emotional Intelligence: A Preliminary Study
Source: PLoS One. 2014 Jul 1;9(7):e101247. doi: 10.1371/journal.pone.0101247 (PMC4077755; doi:10.1371/journal.pone.0101247)
Supplement: Supplementary file 1 [file pone.0101247.s001.doc]

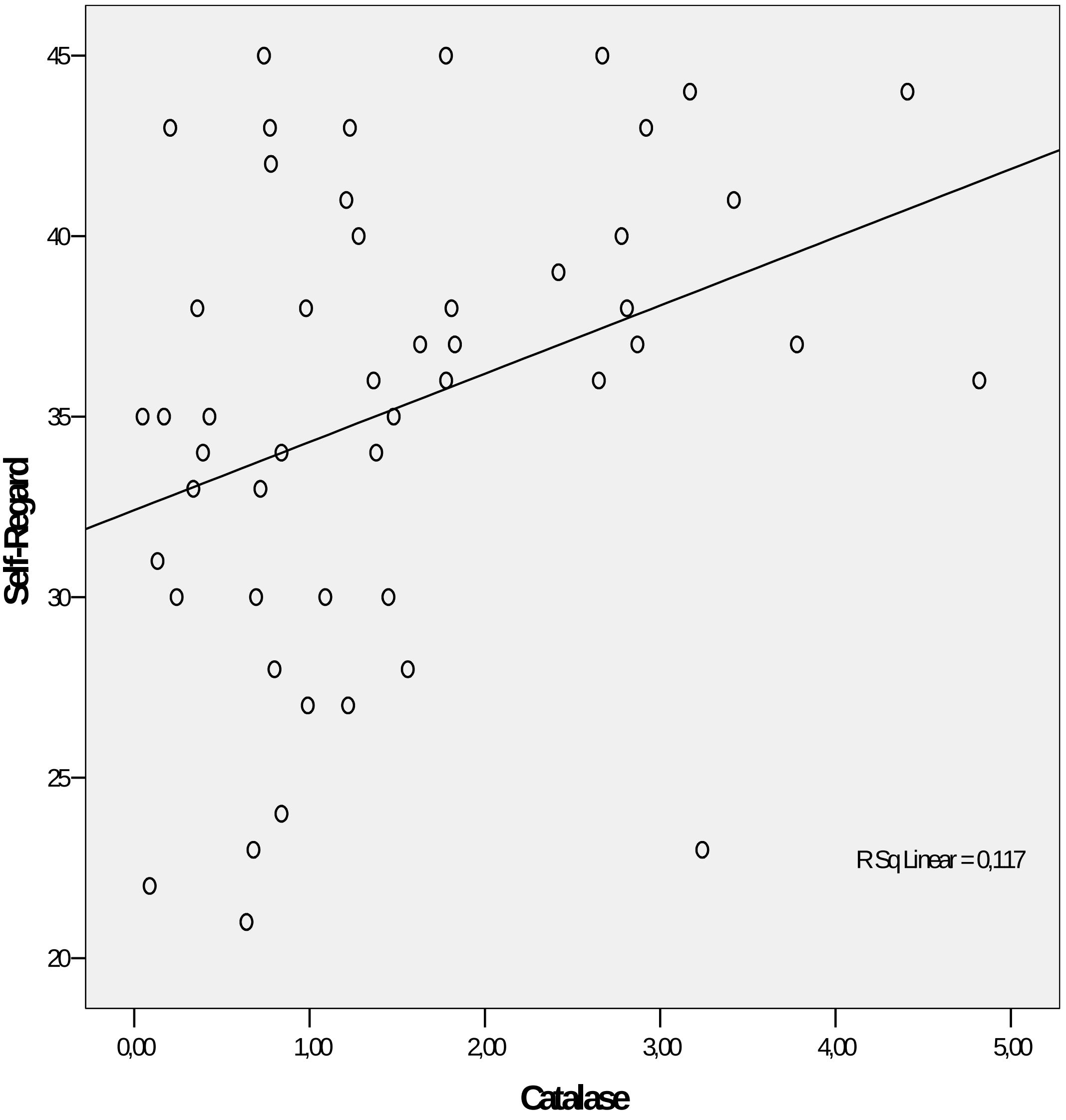

45
40
35
30
25
20
Self-Regard
0,00
1,00
2,00
3,00
4,00
5,00
Catalase
R Sq Linear = 0,117

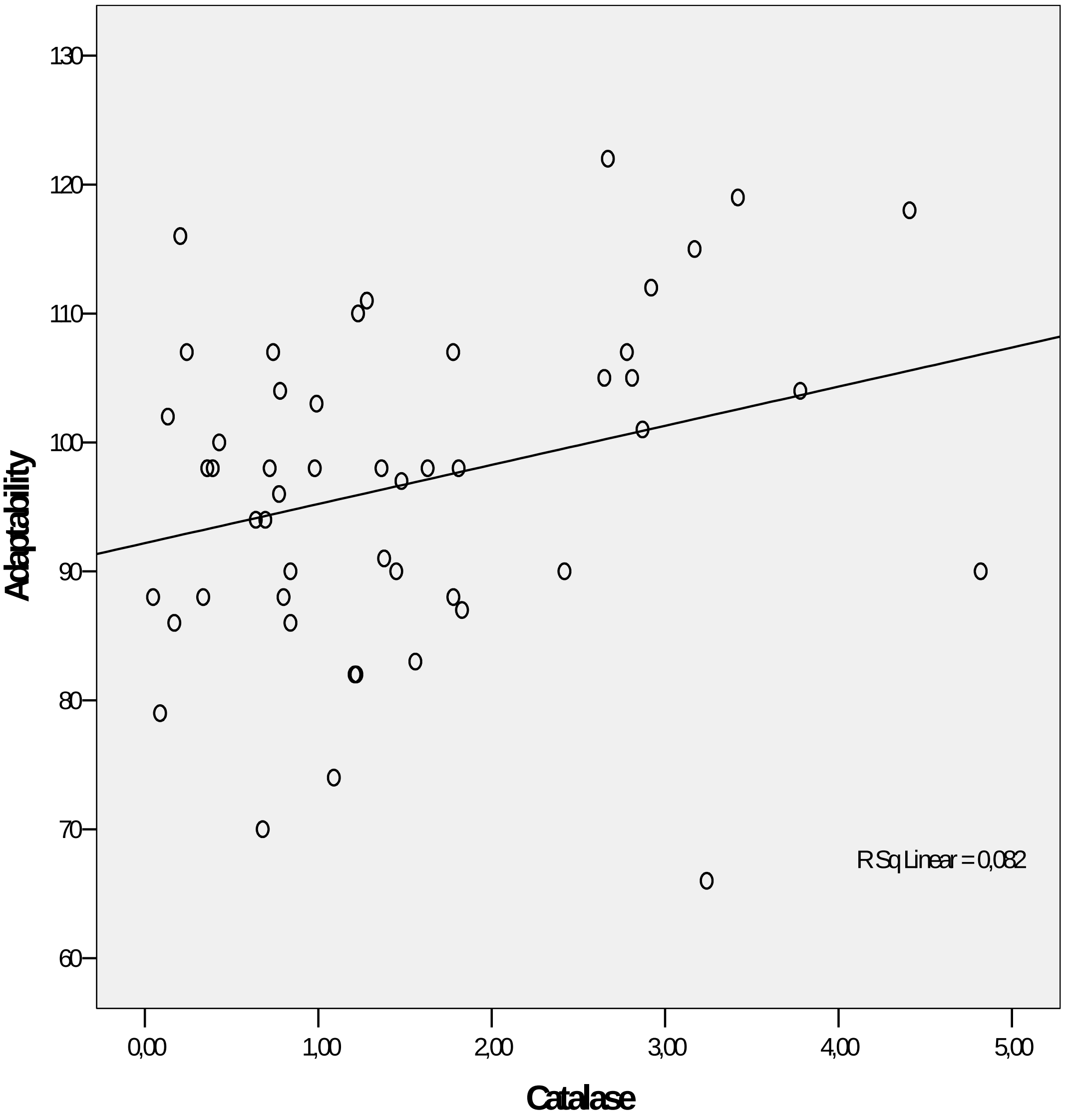
130
120
110
100
90
80
70
60
Adaptability
0,00
1,00
2,00
3,00
4,00
5,00
Catalase
R Sq Linear = 0,082

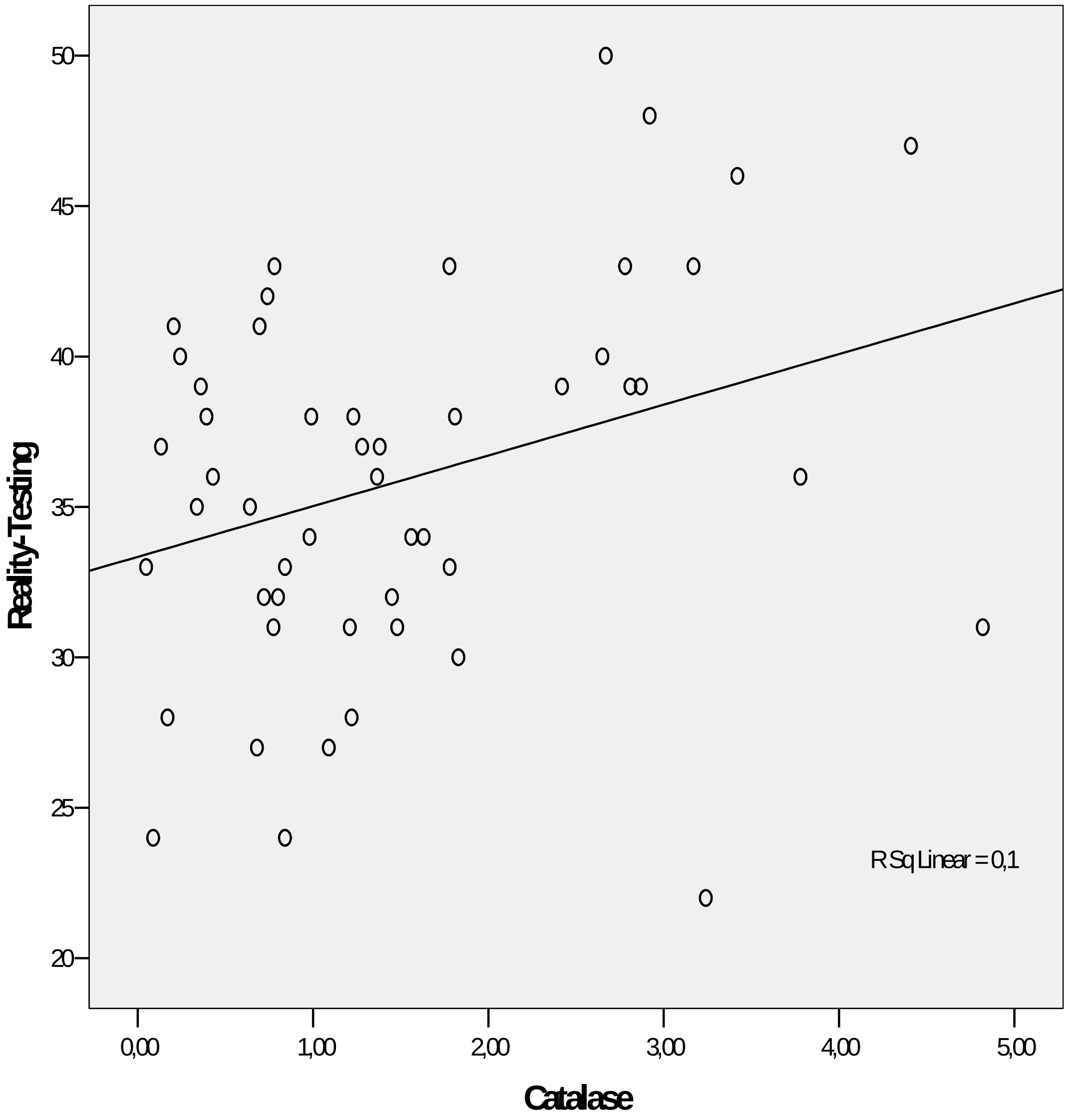

Reality-Testing
50
45
40
35
30
25
20
0,00
1,00
2,00
3,00
4,00
5,00
Catalase
R Sq Linear = 0,1

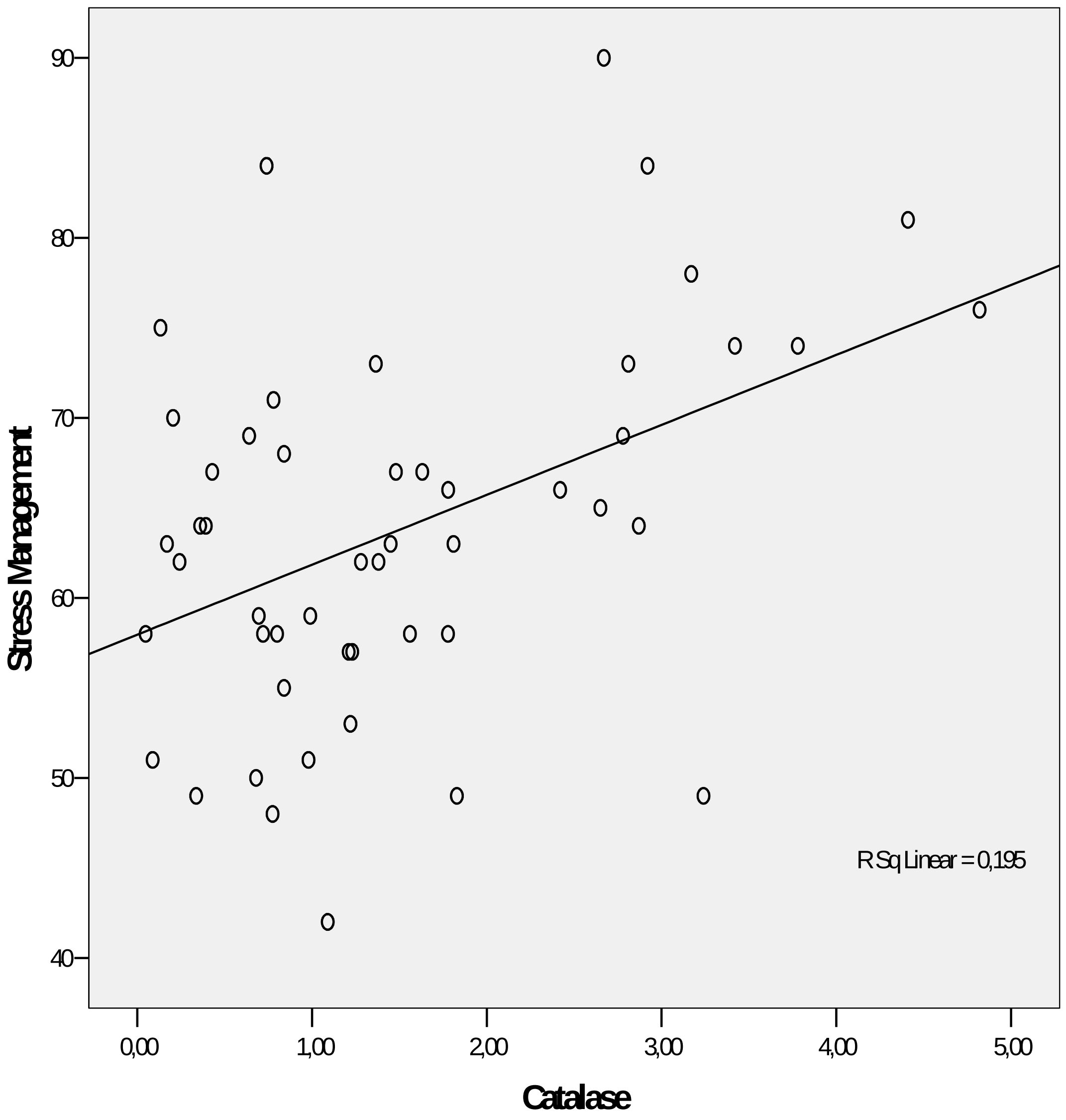
Stress Management
90
80
70
60
50
40
R Sq Linear = 0,195
0,00
1,00
2,00
3,00
4,00
5,00
Catalase

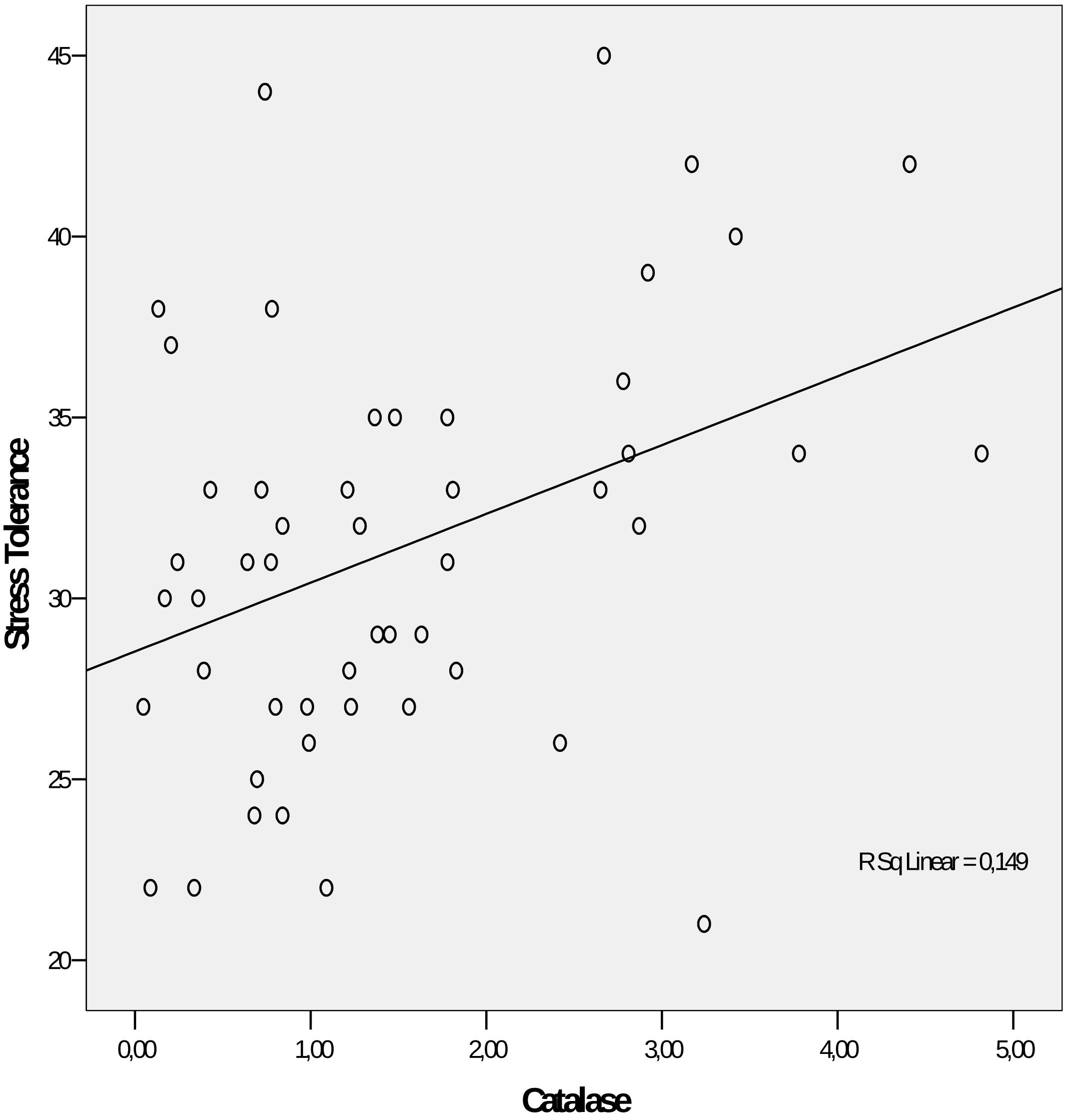

Stress Tolerance
45
40
35
30
25
20
R Sq Linear = 0,149
0,00
1,00
2,00
3,00
4,00
5,00
Catalase

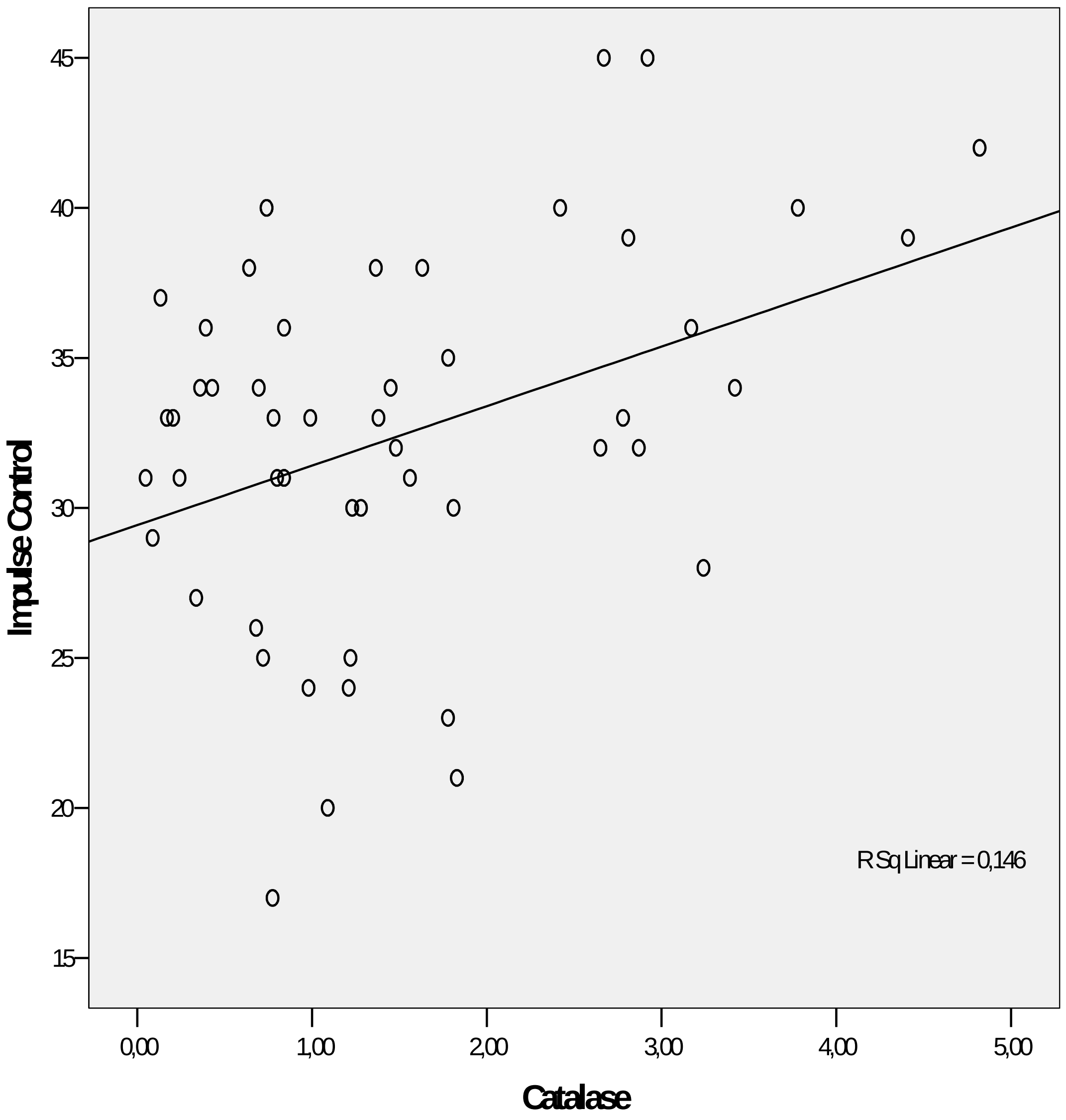

45
40
35
30
25
20
15
Impulse Control
R Sq Linear = 0,146
0,00
1,00
2,00
3,00
4,00
5,00
Catalase

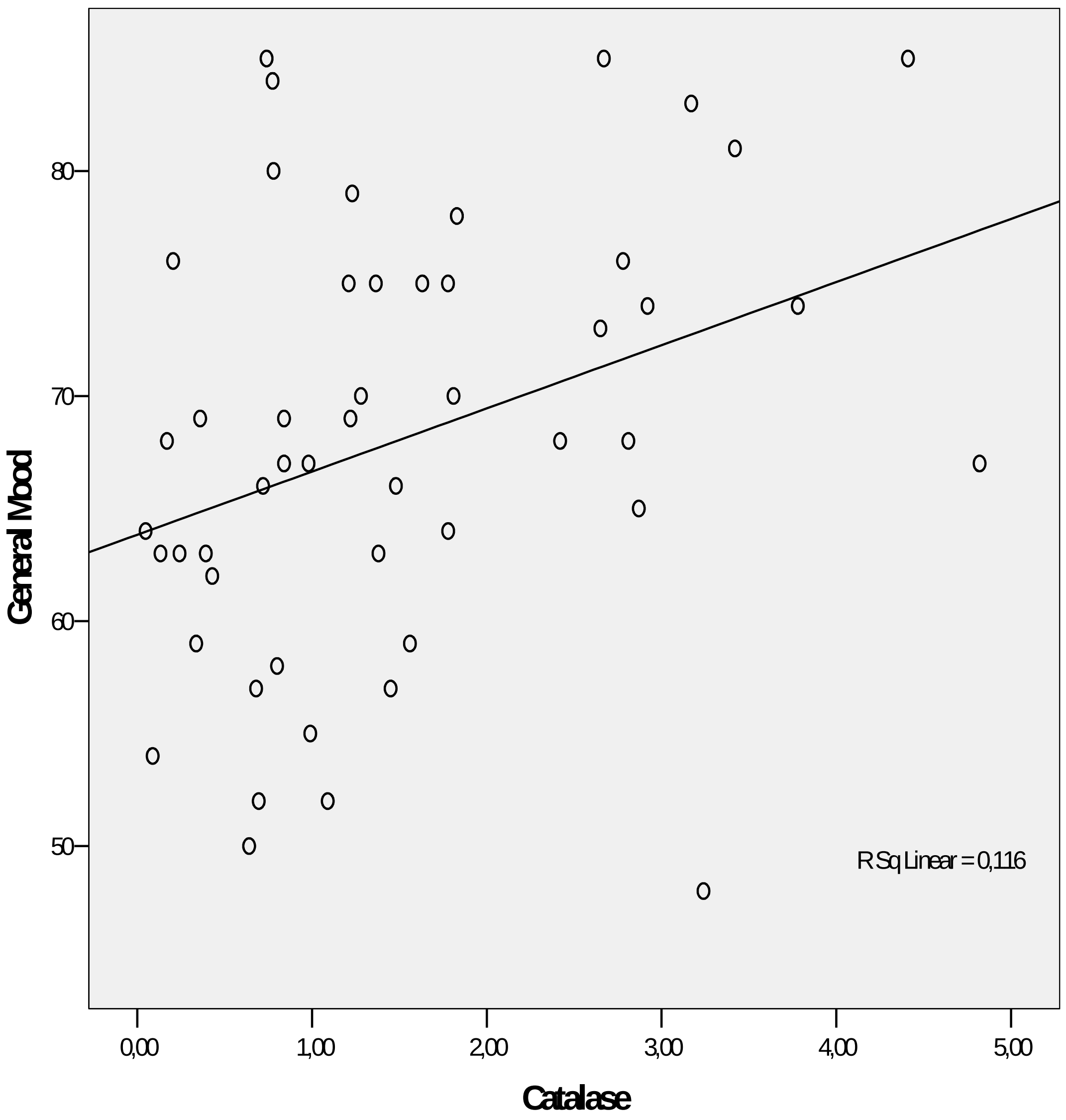

80
70
60
50
General Mood
R Sq Linear = 0,116
0,00
1,00
2,00
3,00
4,00
5,00
Catalase

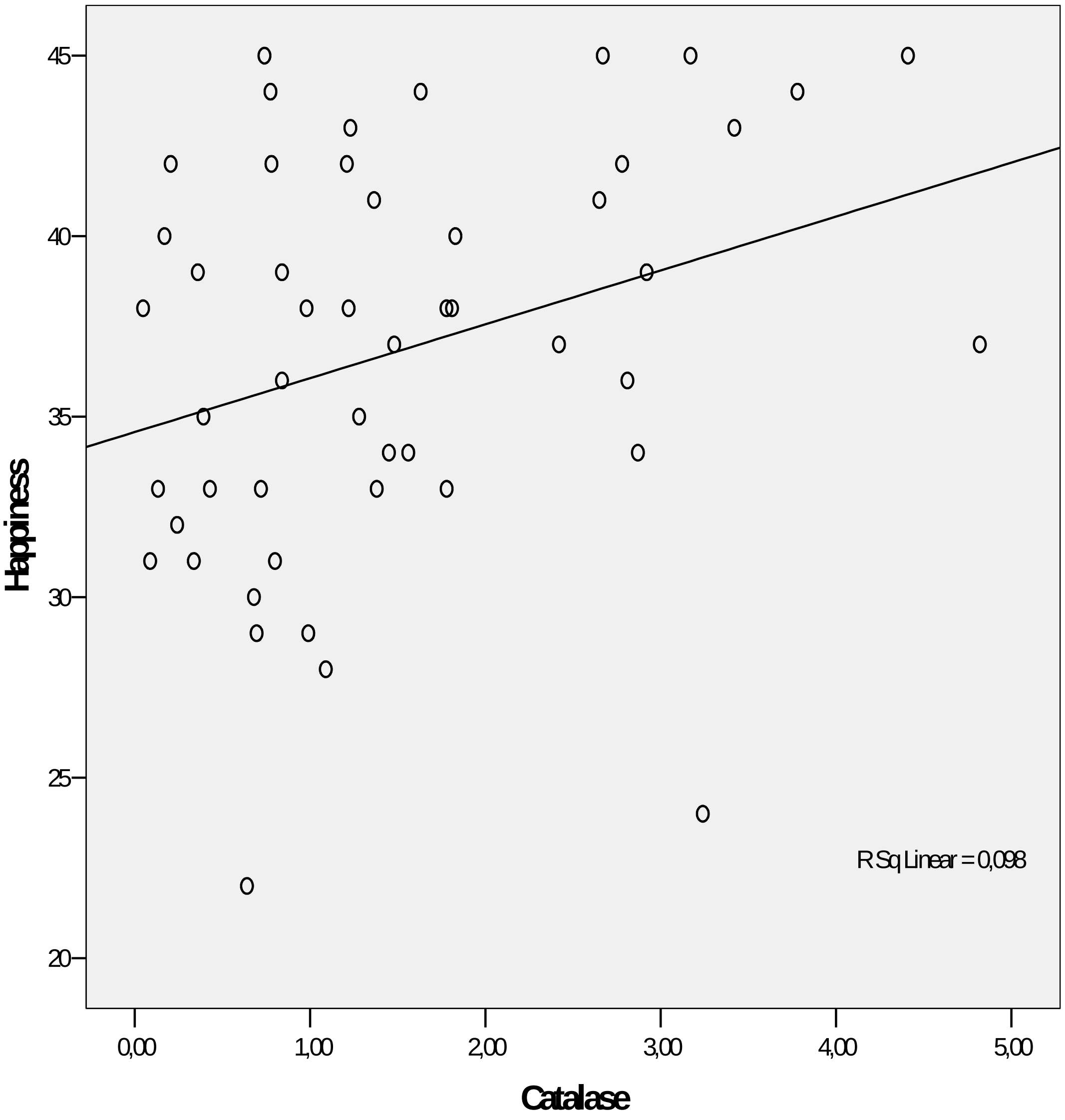

Happiness
45
40
35
30
25
20
R Sq Linear = 0,098
0,00
1,00
2,00
3,00
4,00
5,00
Catalase

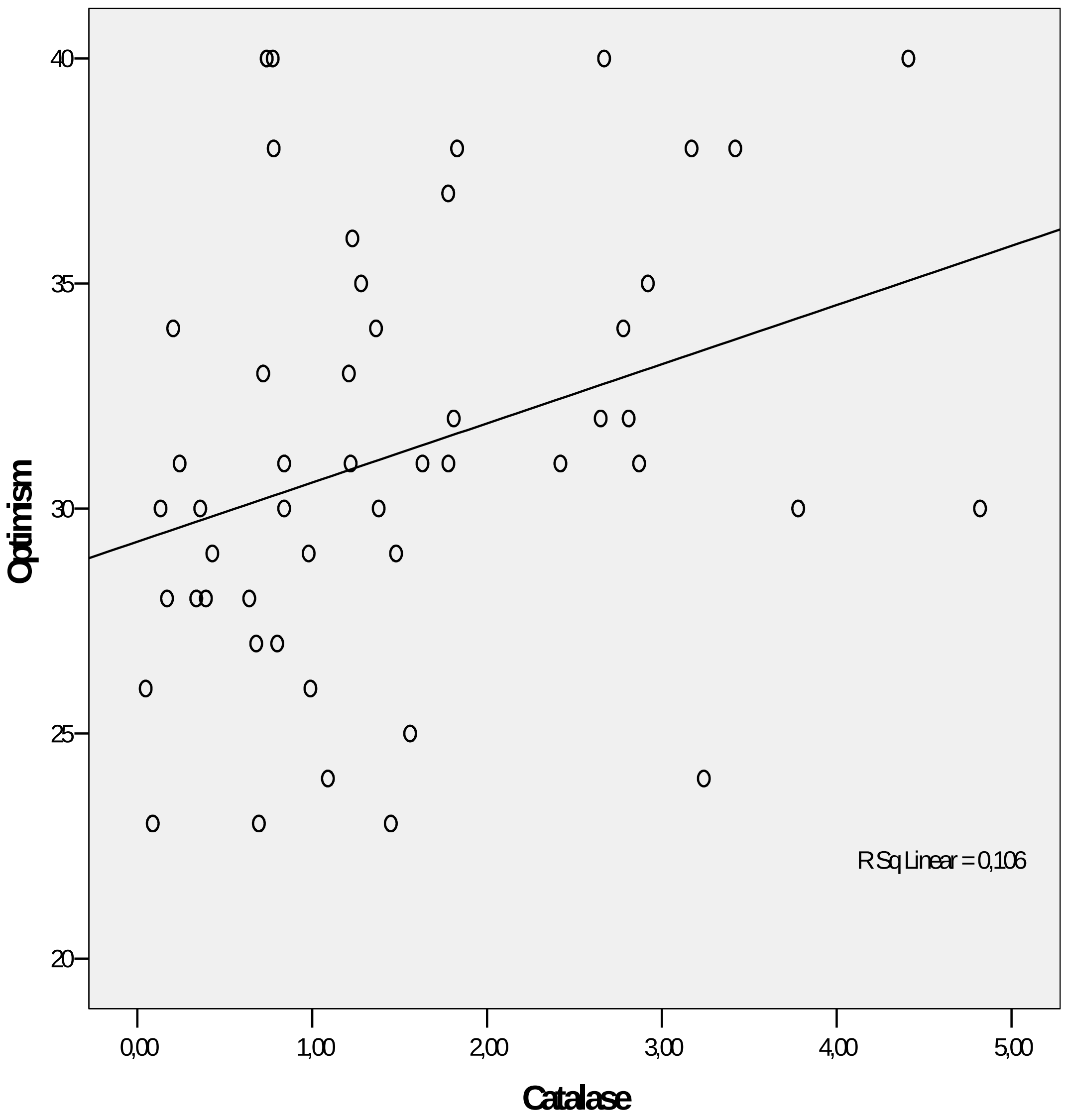

Optimism
40
35
30
25
20
R Sq Linear = 0,106
0,00
1,00
2,00
3,00
4,00
5,00
Catalase

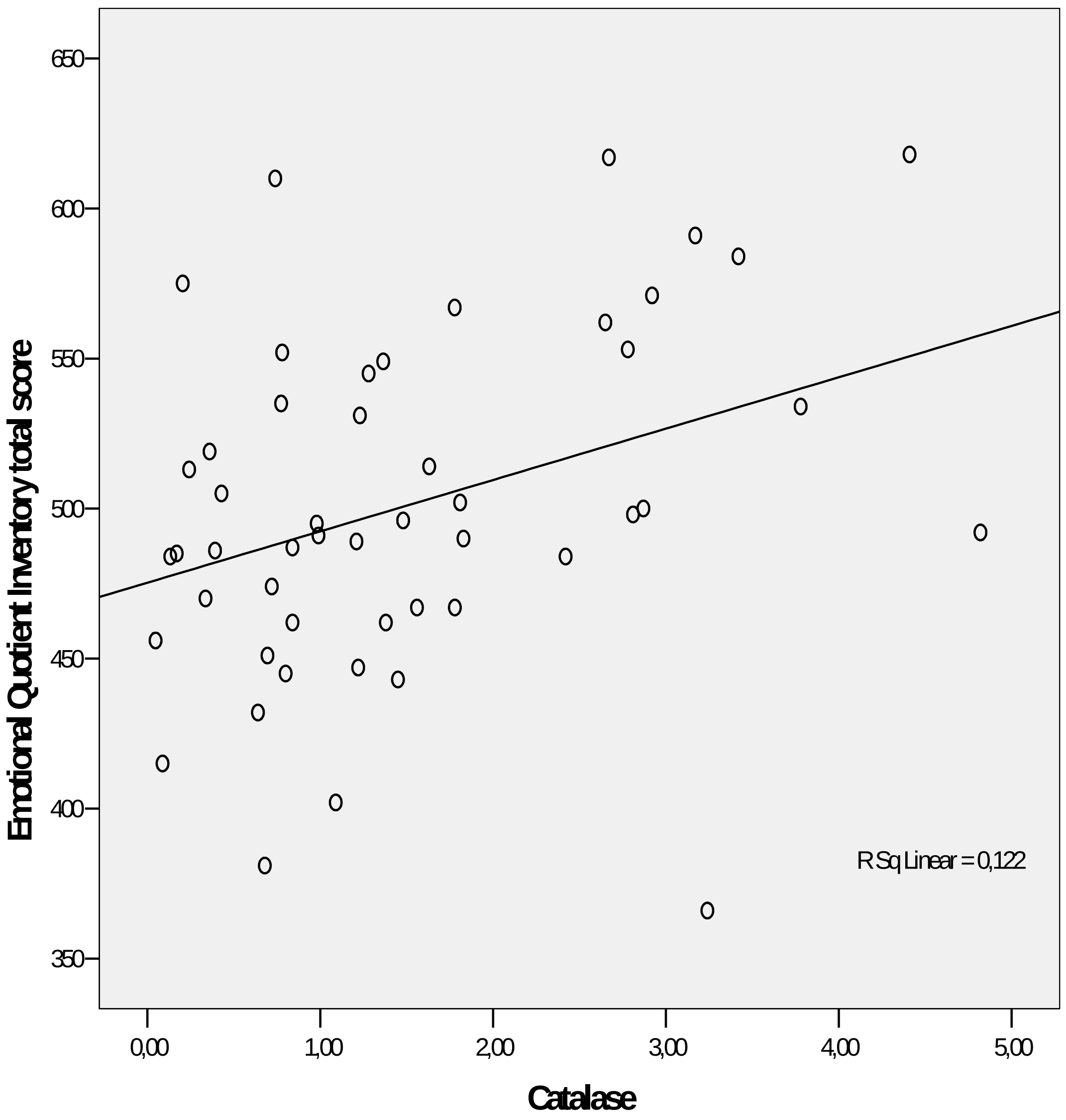
Emotional Quotient Inventory total score
650
600
550
500
450
400
350
0,00
1,00
2,00
3,00
4,00
5,00
Catalase
R Sq Linear = 0,122
